# Prevalence and molecular identification of fish-borne trematodes in endemic communities in Caraga region, Mindanao, Philippines

**DOI:** 10.1016/j.fawpar.2025.e00302

**Published:** 2025-11-17

**Authors:** Vachel Gay V. Paller, Jasmine Renette D. Jimenez, Allen Jethro I. Alonte, Vicente Y. Belizario Jr., Billy P. Divina, Kezia W. Kozel, Martha E. Betson

**Affiliations:** a Institute of Biological Sciences, College of Arts and Sciences, University of the Philippines Los Baños, Philippines; b Department of Parasitology, College of Public Health, University of the Philippines Manila, Philippines; c Department of Veterinary Paraclinical Sciences, College of Veterinary Medicine, University of the Philippines Los Baños, Philippines; d Discipline of Comparative Biomedical Sciences, School of Veterinary Medicine, University of Surrey, Guilford, Surrey, United Kingdom

**Keywords:** Philippines, Caraga region, Fish-borne trematodes, Neglected tropical diseases, *Opisthorchis*

## Abstract

The fish-borne trematodes are a group of parasitic flatworms whose life cycle successively passes through various snails and fresh and brackish water fish, and terrestrial vertebrate hosts including humans. Human infection is common in countries where eating raw fish is practiced such as in the Philippines. Limited investigation in endemic areas along with misdiagnosis makes it challenging to address these infections. Therefore, this study aimed to investigate the prevalence, identify risk factors for, and molecularly identify fish-borne trematodes in eight helminth-endemic communities in Mindanao, Philippines through a household-based cross-sectional study. Fecal samples were collected from 1152 residents (age 10–59 years) from 386 households in the study communities and from 92 dogs and cats. These were examined microscopically for trematode infections. Molecular analysis of microscopy-positive fecal samples was conducted using the internal transcribed spacer 2 region (ITS2) gene. Further, household-heads were surveyed to investigate risk factors for infection.

The overall prevalence of infection in humans was 4.1 % (*n* = 47), with higher infection prevalence in Surigao del Norte province, where fish is often eaten raw or undercooked (as *kinilaw* and *sugba*). Males and adults were found to be more at risk of infection. Only 2 animals were positive, both of which are dogs. Molecular findings revealed three fish-borne trematode species: *Haplorchis taichui*, *Stellanthchasmus falcatus*, and *Opisthorchis viverrini*. To our knowledge, this study reports the first molecular identification of fish-borne trematodes in the Philippines. The results help address knowledge gaps on fish-borne trematodes in the Philippines and can be employed to improve control using evidence-based and targeted approaches.

## 1 Introduction

Fish-borne trematodes (FBTs) are parasitic flatworms parasitizing a wide range of terrestrial vertebrate hosts including humans with fresh- or brackish-water fish as intermediate host. Infection occurs through ingestion of metacercariae lodged in raw or improperly cooked fish (Chai et al., 2009; Mahdy et al., 2021). Improper disposal of fecal waste is the common source of environmental contamination thereby continuing transmission.

Around 7 million people worldwide are believed to be infected with FBTs (World Health Organization, 2021), with most infections found in Southeast Asia including the Philippines, particularly in areas where eating raw food is practiced (Chai et al., 2009; Do et al., 2007). Diseases caused by FBTs are considered as among the most neglected of the neglected tropical diseases. Infection with FBTs may result in various clinical manifestations. Chronic infections lead to symptoms associated with the organ where the adult worm is located. For instance, obstructive jaundice, hepatomegaly are observed in individuals with heavy liver fluke infections, with cholangiocarcinoma a serious secondary complication (Keiser and Utzinger, 2009). In contrast symptoms associated with intestinal flukes are similar to those of peptic ulcer (Belizario et al., 2004). Although usually asymptomatic, migration to other organs (e.g., brain and heart) and even death has been reported in individuals that are heavily infected (i.e., heterophyids). Coinfection with other parasites in human and animal hosts may also occur (Fried and Abruzzi, 2010).

Diagnosis of FBTs is an ongoing problem because of the low sensitivity of microscopy-based methods and the inadequacy of morphological approaches in discriminating parasite eggs, especially within closely related species of superfamily Opisthorchoidea (Do et al., 2007). There has been limited molecular characterization of these parasites in many settings. Diagnostic challenges coupled with low awareness of community and health workers about these parasites means their true burden is underestimated and it is challenging to implement effective control of these helminths (Chai and Jung, 2017; Eduardo, 2001). Thus, this study aimed to investigate the occurrence of FBTs in endemic communities in Caraga Region in Mindanao and identify the parasites through molecular analysis.

## 2 Materials and methods

### 2.1 Study site

The study was conducted as part of the Zoonotic Transmission of Intestinal Parasites (ZooTRIP) project (Kajero et al., 2022) in Caraga Region, specifically in Agusan del Sur and Surigao del Norte which remain endemic for soil transmitted helminthiasis, schistosomiasis, and food borne helminthiasis (Kajero et al., 2022; Mationg et al., 2021; Owada et al., 2018). Four municipalities from each province were selected based on known endemicity of intestinal helminth infections, cooperation of the local government unit, accessibility of the communities, and the security situation.

### 2.2 Sampling design

A household-based sampling was employed, using a cross-sectional mixed method approach. Households were randomly selected from a list of households in each municipality and household members aged 10–59 years were invited to participate. Due to logistical and financial constraints, it was not possible to include all households in the study. Household members with disability (either physical and mental) and pregnant women were excluded from participation in the study. A total of 1152 individuals belonging to 386 households were recruited. Further, companion animals from the selected households were also included to determine possible zoonotic transmission. A total of 92 animals (72 dogs and 20 cats) were included.

### 2.3 Collection and processing of fecal samples

Participants were provided with 60 mL fecal containers and were instructed to collect an approximately 20 g fecal sample. The samples were collected from each household and were transported to field laboratories. For animals, freshly voided fecal samples were collected from dogs and cats present in the household by picking up stools using a spatula from the usual spots where these animals defecate. In cases where samples were unavailable at the time of household visit, the owners were given specimen cups and spatula and were advised to collect freshly excreted stool samples from each animal and submit to the village center the following day. All samples were processed in the field laboratory. Fecal samples from humans were analyzed using the Kato-Katz method (Katz et al., 1972) while animal fecal samples were processed using McMaster technique (Zajac et al., 2012) which are the recommended techniques for the different host populations. Two aliquots were prepared from each sample, and the number of eggs was determined in each aliquot. The average egg count was computed, and the number of eggs per gram (epg) was calculated. All fecal samples were then preserved in absolute ethanol for transport and molecular analysis.

### 2.4 Data processing and analysis

From the results of the parasitological assessment, the parasite prevalence and mean intensity (MI) were calculated. To compare parasite egg prevalence and intensity among locations, sexes, and age categories, a Fisher's exact test was performed at *p*-value ≤0.05 significance. Associations between household level risk factors and presence of at least one infected individual in a household were also assessed using a Fisher's exact test. Analyses were performed using R Statistical Software (v4.2.3) and Stata v14.2.

### 2.5 Molecular identification

Representative positive samples were subjected for species identification through molecular techniques using the ITS2 gene. DNA was extracted from representative positive fecal samples using DNeasy® PowerSoil® Pro (Qiagen, USA) following the manufacturer's protocol. Pre-extraction was carried out by homogenizing the stool sample in the kit's zirconium bead tube for three minutes using a beadbeater (Benchmark D2400-E BeadBlaster). DNA was labelled and stored at −20 °C until further use. A standard polymerase chain reaction was performed to amplify the ITS2 region of the parasite. This was selected as it is highly conserved at species level and allows characterization of digeneans such as liver flukes and minute intestinal flukes, at different category levels (Waikagul and Thaenkham, 2014). This region was amplified using previously published primers (Sato et al., 2010).

PCR amplification was carried out using 3.125 μL of PCR master mix (1st Base exTEN ® 2×), 0.5 μM primers, 1 μL DNA template, and nuclease-free water to reach a total reaction volume of 25 μL. Negative controls (nuclease-free water) were included in each run. A standard PCR was run using the amplification profiles previously described (Sato et al., 2010). All samples showing expected band sizes (330–530 bp) in gel electrophoresis were confirmed through sequencing. Forward and reverse amplicon sequencing using the Sanger method was outsourced to Apical Scientific Laboratory (Selangor, Malaysia). Sequences generated were submitted to GenBank (PQ818165-PQ818167).

### 2.6 DNA sequence analysis and phylogenetic tree construction

Consensus sequences were generated from good quality sequences for both forward and reverse runs, otherwise, only the good quality run (either forward or reverse) was used. Consensus sequences were built using MEGA X software by aligning the forward sequence and the reverse sequence (reverse complemented). A BLAST search (National Center of Biotechnology Information public databases; http://blast.ncbi.nlm.nih.gov/Blast.cgi) was done using the generated consensus sequences to identify most closely matching sequences in the database. Multiple sequence alignment of the nucleotide sequences was performed using the Clustal W algorithm in MEGA X software with all the parameters set to default. The alignments were visually inspected, and leading and trailing misaligned regions were trimmed to minimize the effects of sequence length in the phylogenetic analysis.

Phylogenetic trees were reconstructed in MEGA X with the distantly related trematode – *Echinostoma miyagawai* (MH796365) - used as outgroup. The best nucleotide substitution model was identified and the substitution model with the lowest corrected Bayesian Information Criterion (BIC) was selected. General Time Reversible model was used as the nucleotide substitution model with gamma distribution set at 0.7046 as shape parameter. A maximum likelihood tree was constructed following the parameters set by the model identified. A bootstrap consensus tree was built with 5000 bootstrap replicates and branches corresponding to partitions reproduced in less than 50 % bootstrap replicates were collapsed. Furthermore, Bayesian inference of phylogeny was computed through determining the posterior probability (BPP). This was done using the Markov Chain Monte Carlo (MCMC) algorithm in MrBayes (v3.2). Parameters were set following the best substitution model previously identified. MCMC was run for 1 million generations sampling for every 100th generation and setting 250,000 (25 % of the generations) as burn in. The number of generations were set to ensure convergence of the model with split frequency value of <0.01.

## 3 Results

### 3.1 Prevalence and intensity

A total of 1152 participants belonging to 386 households were surveyed. Overall, 47 individuals (4.1 %) were found positive for FBT, with a mean egg count of 214 epg. Six individuals had an FBT egg count of greater than 500 epg, ranging from 504 to 1944 epg. A higher parasite prevalence was observed in the municipalities of Mainit and Claver, Surigao del Norte (*p* < 0.001). Higher mean intensity was observed in Esperanza, Agusan del Sur. Furthermore, more males (6.4 %) were found to be infected than females (2 %) (p < 0.001), while more adults were infected (5.5 %) than children (2 %) (p < 0.001) (Table 1). In animals, out of 92 animal fecal samples examined, only two animal fecal samples (2 %) were positive: specifically, one dog from Trento in Agusan del Sur and one dog from Mainit, Surigao del Norte. Of 386 households included in the study, 25 had at least one infected individual infected with FBT. Analysis of risk factors at household level did not reveal an association with educational level of household head, occupation of the father or mother or reported consumption of raw or undercooked food within the household (Table 2).

**Table 1:** Prevalence and mean intensity of fish-borne trematode infections detected using Kato-Katz analysis of human fecal samples from Northeastern Mindanao (*n* = 1152).

Variable	Category	No. examined	No. positive (%)	Confidence Interval (95 %)	Mean Intensity (epg)	Standard Deviation
Municipality in Agusan del Sur Province	Bunawan	168	1 (0.6)	0.0–1.8	456	–
	Trento	145	6 (4.1)	1.0–7.4	396	482
	Bayugan	120	1 (0.8)	0.0–2.5	36	–
	Esperanza	133	2 (1.5)	0.0–3.6	984	1358
	S*ubtotal*	566	10 (1.8)	0.7–2.9	484	646
Municipality in Surigao del Norte Province	Mainit	181	16 (8.8)	4.7–13.0	240	293
	San Isidro	169	0	–	–	–
	Claver	126	14 (11.1)	5.6–16.6	88	87
	Gigaquit	110	7 (6.4)	1.8–10.9	24	12
	*Subtotal*	586	37 (6.3)	2.9–5.2	142	216
Sex	Male	548	35 (6.4)	4.3–8.4	187	349
	Female	604	12 (2.0)	1.0–3.1	295	439
Age	Child (10–17)	532	12 (2.3)	1.0–3.5	221	381
	Adult (18+)	620	35 (5.7)	3.8–7.5	196	357
Overall		1152	47 (4.1)	3.0–5.4	214	372

**Table 2:** Household level prevalence of fish-borne trematode infections and household-level risk factors.

Variable	Category	No. examined	No. positive (%)	Confidence Interval (95 %)	p-value
Education level of household head	No schooling	1	0	–	0.261
	Elementary school	140	13 (9.3)	5.0–15.4	
	High school	186	10 (5.4)	2.6–9.7	
	College education	47	1 (2.1)	0.05–11.3	
	Vocational	4	0	–	
Father's occupation	Unemployed	24	1 (4.2)	0.1–21.1	0.137
	Farming	151	14 (9.3)	5.2–15.1	
	Other	116	3 (2.6)	0.5–7.3	
	Two/more jobs	7	0	–	
Mother's occupation	Unemployed	163	9 (5.5)	2.6–10.2	0.261
	Farming	28	1 (3.6)	0.09–18.3	
	Laundry	25	1 (4.0)	0.1–20.4	
	Other	103	1 (1.0)	0.02–5.3	
	Two/more jobs	2	0	–	
Consumption of raw/undercooked food	No	276	21 (7.6)	4.8–11.4	0.271
	Yes	89	3 (3.4)	0.7–9.5	

### 3.2 Molecular analysis

Representative positive samples from both humans and animals were further subjected to molecular analysis. In humans, two different band sizes were observed: ∼350 bp and ∼ 480 bp. Amplicons of each size from these samples were submitted for sequencing. Unfortunately, no DNA was amplified from the two animal samples that were found positive for FBT by microscopy.

The resulting ITS sequences of the 350 bp amplicons had 98.3 % identity with *Haplorchis taichui* (Accession no. MF043945; 6 sequences), with 100 % query coverage and an *E*-value of 4.0e-105. The ITS sequences of the ∼380 bp amplicons showed 99.1 % identity with *Opisthorchis viverrini* (Accession no. MT002731; 5 sequences) with 100 % query coverage and an E-value of 1.0e-157 or 94.6 % identity with *Stellantchasmus falcatus* (Accession no. KJ630833; 2 sequences) with 100 % query coverage and an E-value of 1.0e-158. Variable sites identified on alignment of sequences generated with representative sequences of *H. tachui*, *O, viverrini* and *S. falcatus* available on Genbank are summarized in Table 3. All sequences identified as *H. taichui* were from Mainit, Surigao del Norte, while the 5 sequences identified as *O. viverrini* were from Trento (3) and Bunawan (1), Agusan del Sur and Mainit, Surigao del Norte (1). Further, the two *S. falcatus* sequences were from Surigao del Norte particularly in Claver and Gigaquit (Table 4 and Table 5).

**Table 3:** Variable sites identified on alignment of ITS2 sequences generated in this study and ITS2 sequences for FBTs available on Genbank.

Species	VDNA Position⁎																													
*H. taichui*	77	130	205	220	231	233	235	236	237	238	239	241	242	247	248	249	251	252	253	256	257	258	259	260	261	263	267	270	272	273
This study	T	C	T	A	A	A	–	–	–	–	–	C	A	A	A	T	C	T	C	–	–	–	–	–	–	G	C	T	C	T
MF043945	A	–	C	G	T	G	T	G	A	G	G	A	G	G	G	C	G	G	T	G	T	G	G	C	T	A	A	A	T	G
***O. viverrini***	**59**	**94**	**162**																											
This study	G	G	T																											
MT002731	–	–	C																											
***S. falcatus***	**21**	**126**	**128**	**180**	**184**	**199**	**210**	**238**	**241**	**272**	**292**	**312**	**317**	**318**	**319**	**334**	**343**	**355**	**359**	**370**										
This study	T	A	G	A	A	–	G	A	G	T	C	T	–	–	–	C	T	G	C	T										
KJ630833	C	T	A	G	G	T	A	G	A	A	T	C	A	T	T	G	C	–	A	A										

⁎ *based on the aligned and trimmed sequences (excluding leading and trailing regions).*

**Table 4:** Molecular identification using ITS2 gene of fish-borne trematodes from human fecal samples from Northeastern Mindanao.

Province	Municipality	*Haplorchis taichui*	*Opistorchis viverrini*	*Stellantchasmus falcatus*
Agusan del Sur (ADS)	Bunawan	–	1	–
	Trento	–	3	–
	S*ubtotal*	–	**4**	–
Surigao del Norte (SDN)	Mainit	6	1	–
	Claver	–	–	1
	Gigaquit	–	–	1
	*Subtotal*	**6**	**1**	**2**
Overall		**6**	**5**	**2**

**Table 5:** Kato Katz vs PCR results.

Sample No.	Province	Egg counts (epg)	PCR (band size)	Identity (sequencing)
1	Agusan del Sur	456	∼380 bp	*O. viverrini*
2	Agusan del Sur	192	∼380 bp	*O. viverrini*
3	Agusan del Sur	96	*no bands*	
4	Agusan del Sur	120	∼380 bp	*O. viverrini*
5	Agusan del Sur	696	∼380 bp	*O. viverrini*
6	Surigao del Norte	96	*no bands*	
7	Surigao del Norte	480	*no bands*	
8	Surigao del Norte	72	*no bands*	
9	Surigao del Norte	1080	∼530 bp	*H. taichui*
10	Surigao del Norte	24	*no bands*	
11	Surigao del Norte	144	∼530 bp	*H. taichui*
12	Surigao del Norte	48	*no bands*	
13	Surigao del Norte	216	∼530 bp	*H. taichui*
14	Surigao del Norte	624	∼380 bp	*O. viverrini*
15	Surigao del Norte	240	∼530 bp	*H. taichui*
16	Surigao del Norte	24	∼530 bp	*H. taichui*
17	Surigao del Norte	24	∼530 bp	*H. taichui*
18	Surigao del Norte	24	*no bands*	
19	Agusan del Sur	36	*no bands*	
20	Surigao del Norte	24	*no bands*	
21	Surigao del Norte	84	*no bands*	
22	Surigao del Norte	48	*no bands*	
23	Surigao del Norte	312	∼380 bp	No good sequencing reads
24	Surigao del Norte	96	*no bands*	
25	Surigao del Norte	60	*no bands*	
26	Surigao del Norte	132	∼380 bp	*S. falcatus*
27	Surigao del Norte	36	*no bands*	
28	Surigao del Norte	12	*no bands*	
29	Surigao del Norte	36	*no bands*	
30	Agusan del Sur	24	*no bands*	
31	Agusan del Sur	24	*no bands*	
32	Surigao del Norte	24	∼380 bp	*S. falcatus*
33	Surigao del Norte	24	*no bands*	

**Fig. 1:**
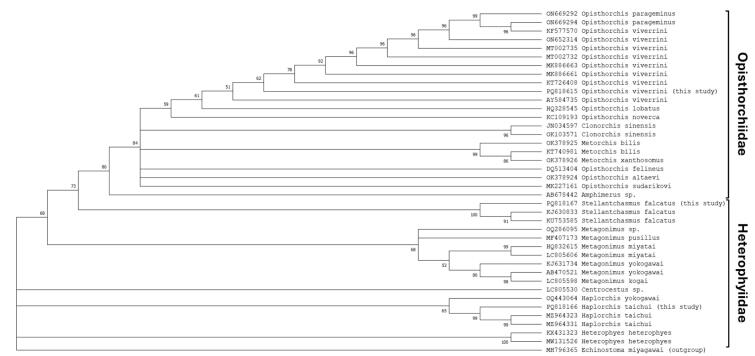
Phylogenetic tree of trematodes based on the ITS2 gene. Bootstrap support values are indicated in each branch. Branch values lower than 50 % were collapsed.

The generated ITS2 sequences were aligned with previously published sequences from NCBI databases using the CLUSTAL W algorithm of MEGA X. A total of 40 sequences were aligned including three unique sequences generated in this study and the outgroup *E. miyagawai* (MH796365). Fig. 1 shows the phylogenetic tree based on ITS2 genes of different trematodes reconstructed using the aligned sequences and the maximum likelihood method. The generated tree showed a paraphyletic clade labelled grouping the members of the family Opistorchiidae with bootstrap value of 80 %. Members of this group include the genus *Opisthorchis* spp., *Chlonorchis* sp., *Metorchis* spp., and *Amphimerus* sp. It can be noted that the sequence generated in this study grouped well with other *O. viverrini* sequences. Furthermore, the family Heterophyiidae did not form a distinct clade but lies outside the paraphyletic clade of the Opistorchiidae. Each genus under the family Heterophyiidae has a separate grouping which include the genera *Stellantchamus* sp.*, Metagonimus* spp., *Centrocestus* sp., *Haplorchis* spp., and *Heterophyes* spp. Sequences generated in this study grouped with the expected genus. A phylogenetic tree constructed using Bayesian inference showed a similar overall topology (Supplementary Fig. 1).

## 4 Discussion

In this study we demonstrated the presence of FBT infections in humans living in seven communities in Northeastern Mindanao, the Philippines. To our knowledge, this is the first study to report molecular identification of FBTs circulating in the country.

The study found a relatively low overall infection rate of FBT in humans compared with surveys done in other areas in Mindanao (Belizario et al., 2022; Belizario et al., 2004; Belizario et al., 2001; Moendeg et al., 2021). Reasons for this could include the low sensitivity of the Kato Katz and other microscopy-based techniques for detection of FBT infections and potential egg degradation prior to diagnosis (Johansen et al., 2015). Nevertheless, it was observed there were pockets of endemicity in particular geographic areas. Access to nearby fishing sites or sources of fishes, and cultural practices such as consumption of infected fish predispose communities in acquiring FBTs (Chai and Jung, 2017; Moendeg et al., 2021). There appeared to be a higher risk of acquiring the infection in Surigao del Norte. Infection occurs in three municipalities in Surigao del Norte: Mainit, Claver and Gigaquit, while no FBT infection was found in San Isidro, which is located on a separate island. Surigao del Norte is a fish-producing region, with Lake Mainit, the fourth largest lake in the Philippines and a valuable fishery resource (Biña-De Guzman et al., 2013), situated in the province. Meanwhile, the highest burden of FBT infection in Agusan del Sur was observed in Trento. Only six individuals showed a high FBT infection burden based on fecal egg counts– which could be due to habitual consumption of raw or inadequately cooked fish, as well as visit in other areas that are endemic with FBTs. Increased mobility and distribution of fish sold outside the local community (Keiser and Utzinger, 2009; Phan et al., 2011) could also contribute to the spread of infection in non-endemic areas. There is also a wide range of marine and freshwater fish hosts of these trematodes (Clausen and Murrell, 2014; Romero-Alvarez et al., 2020). In addition, pickling or salting the fish may cause possible infection in non-endemic areas because of exportation of fish. Therefore, spatial distribution of these parasites may increase in the future. It should be noted that study sites were selected based on defined criteria as described in the Materials and Methods rather than randomly. This may have introduced bias in the overall estimation of prevalence.

In terms of sociodemographic factors, males and older individuals were found to have higher infection rates than females and young individuals. Previous studies found that social factors affect habits of eating raw fish. For instance, fishermen in Vietnam tend to share and to enjoy raw fish together, as noted by Phan et al. (2011), adding that people enjoyed consuming raw fish with alcohol as a way to socialize (Phan et al., 2011). Similarly, males in the Philippines prefer to eat *kinilaw* together with alcoholic drinks as a form of relaxation (Moendeg et al., 2021). Meanwhile, the lower prevalence in younger individuals could be associated with lower preference of children for raw foods, or due to the cumulative exposure of adults to the parasite over a long time-period to through consumption of raw or undercooked fish. Araki and colleagues noted that consumption of raw fish among children in Laos depends on the mother's preference for raw fish dishes, but it is also possible that the mother will prevent her child to eat raw fish if she thinks it will cause harm, thus lowering the risk of acquiring FBT infection among children (Araki et al., 2018). In our study, we did not observe any association between consumption of raw or undercooked food and FBT infection at a household level. This might be due to the low number of infected households together with the low number of households reporting consumption of raw or undercooked food. Similarly, no associations were observed between FBT infection and educational attainment of the head of household, father's occupation and mother's occupation.

The differences in the pathology and severity of FBT infections make identifying them even to genus level a necessity during surveillance studies. However, identifying FBTs to genus is not feasible by microscopy and requires molecular identification. FBTs are generally reported as heterophyids in the Philippines as this group has a morphologically similar egg and recovering adults are proven to be difficult. This study utilized the nuclear DNA ITS2 marker to identify the eggs seen during microscopy and three species were identified namely *Haplorchis taichui, Stellanthchasmus falcatus,* and *Opisthorchis viverrini*. These FBTs were previously reported in the Philippines with *H. taichui* being the most commonly identified (Belizario et al., 2004; Leonardo et al., 2008). *Stellanthchasmus falcatus* has been reported in the Philippines (Eduardo, 2001) and there has been one opisthorchiasis case report, where the parasite was recovered during a surgical operation of the bile ducts (Belizario Jr and Ciro, 2020). These identifications were based on the recovery of adult fluke. To our knowledge, this study is the first to confirm through molecular analysis the identity of these FBTs. Our results demonstrate the utility of the ITS marker for identifying FBTs in fecal samples, but also highlight some limitations. PCR performance may be affected by low egg counts and inhibitors in stool (Teimoori et al., 2019), and several sequences showed lower than expected similarity for a highly conserved region. This may reflect gaps in GenBank reference data for heterophyids, degraded DNA, or mixed-species templates (Santos and Borges, 2020). We selected ITS as our primary marker because it amplifies reliably and has been widely and successfully applied in trematode identification (Le et al., 2017; Mulyani and Rosidah, 2022). Nonetheless, complementary markers could strengthen species resolution: 28S rDNA is valuable for higher-level systematics but may not reliably distinguish morphologically similar heterophyids (Martínez-Aquino et al., 2019), while mitochondrial loci such as *cox1* offer finer resolution yet are constrained by incomplete and uneven reference data (Santos and Borges-Fernandes, 2020).

Future work on FBTs in the Philippines should include examination of local fish species that are eaten raw or lightly prepared and exploration of snail intermediate hosts to elucidate transmission routes, which are essential in formulating possible preventive measures. In terms of consumption of fish, studies on the viability of metacercariae in different food preparation procedures such as salting or pickling, season of eating, and source of fish would contribute to building sustainable preventive measures especially in communities where eating raw fish is culturally-rooted. Furthermore, recovery of adult worms from the study areas is recommended to connect both morphological and molecular data for Philippine isolates. Finally, the use of other markers such as cytochrome *c* oxidase subunit 1 (*cox1*) and NADH dehydrogenase subunit 1 (*nad1*) to study the population genetics of *O. vierinni* could provide insights into the emergence of this parasite in the Philippines which may have resulted from population migration with the southeast Asia region (Buathong et al., 2020; Pitaksakulrat et al., 2018; Sota et al., 2022; Suwannahitatorn et al., 2024).

## 5 Conclusion

Fish-borne trematodiases remain among the most neglected of the neglected tropical diseases. This study demonstrated that fish-borne trematode infections are circulating in rural areas in Agusan del Sur and Surigao del Norte in Mindanao, Philippines. Through the first molecular identification of FBTs in the Philippines, the presence of *H. taichui*, *S. falcatus*, and *O. viverrini*, were identified which could provide important information to guide effective control and treatment.

Overall, the struggle to eradicate FBTs and other intestinal parasites results from the biosocial complexities of transmission and poor recognition of infection status and parasite distribution. Although molecular-based techniques can now be applied in diagnosis and surveillance, the high cost and accessibility to facilities makes it unlikely to become a standard tool for routine diagnosis especially in rural areas. Nonetheless, adapting site-appropriate prevention and intervention strategies based on research is a step closer to the control of these neglected diseases.

## Ethics approvals

This study was reviewed and approved by the University of the Philippines Manila Research Ethics Board (UPMREB 2019–084-01), the University of Philippines Los Baños Institutional Animal Care Use for Research Committee (CAS 2018–020), the University Ethics Committee of University of Surrey (UEC 2019 049), and the Animal Welfare and Ethical Review Board of the University of Surrey (OUT036). Consent and assent forms were secured and voluntary participation and withdrawal from the study at any time without implication were also ensured. Unique codes were assigned to collected samples to ensure anonymity and confidentiality.

## Funding

This work was supported by the Department of Science and Technology-10.13039/501100011096Philippine Council for Health Research and Development (DOST-PCHRD) and 10.13039/501100000265Medical Research Council (MRC) under a NEWTON-AGHAM grant. Also, partially funded by the DOST-Accelerated Science and Technology Human Resource Development Program (DOST-ASTHRDP) through a thesis grant to the second author.

## CRediT authorship contribution statement

**Vachel Gay V. Paller:** Writing – review & editing, Writing – original draft, Supervision, Funding acquisition, Conceptualization. **Jasmine Renette D. Jimenez:** Writing – original draft, Methodology, Investigation. **Allen Jethro I. Alonte:** Writing – review & editing, Writing – original draft, Methodology, Investigation. **Vicente Y. Belizario Jr.:** Writing – review & editing, Conceptualization. **Billy P. Divina:** Writing – review & editing, Investigation, Conceptualization. **Kezia W. Kozel:** Writing – review & editing, Methodology, Investigation. **Martha E. Betson:** Writing – review & editing, Supervision, Funding acquisition, Conceptualization.

## Declaration of competing interest

The authors declare no conflict of interest.

## Data Availability

To request access to data supporting this study, please contact vvpaller@up.edu.ph or m.betson@surrey.ac.uk.

## Appendix A Supplementary data

Supplementary material Image 1
