# Supplementary figures and images for: Prevalence and molecular identification of fish-borne trematodes in endemic communities in Caraga region, Mindanao, Philippines
Source: Food Waterborne Parasitol. 2025 Nov 17;41:e00302. doi: 10.1016/j.fawpar.2025.e00302 (PMC12681874; doi:10.1016/j.fawpar.2025.e00302)

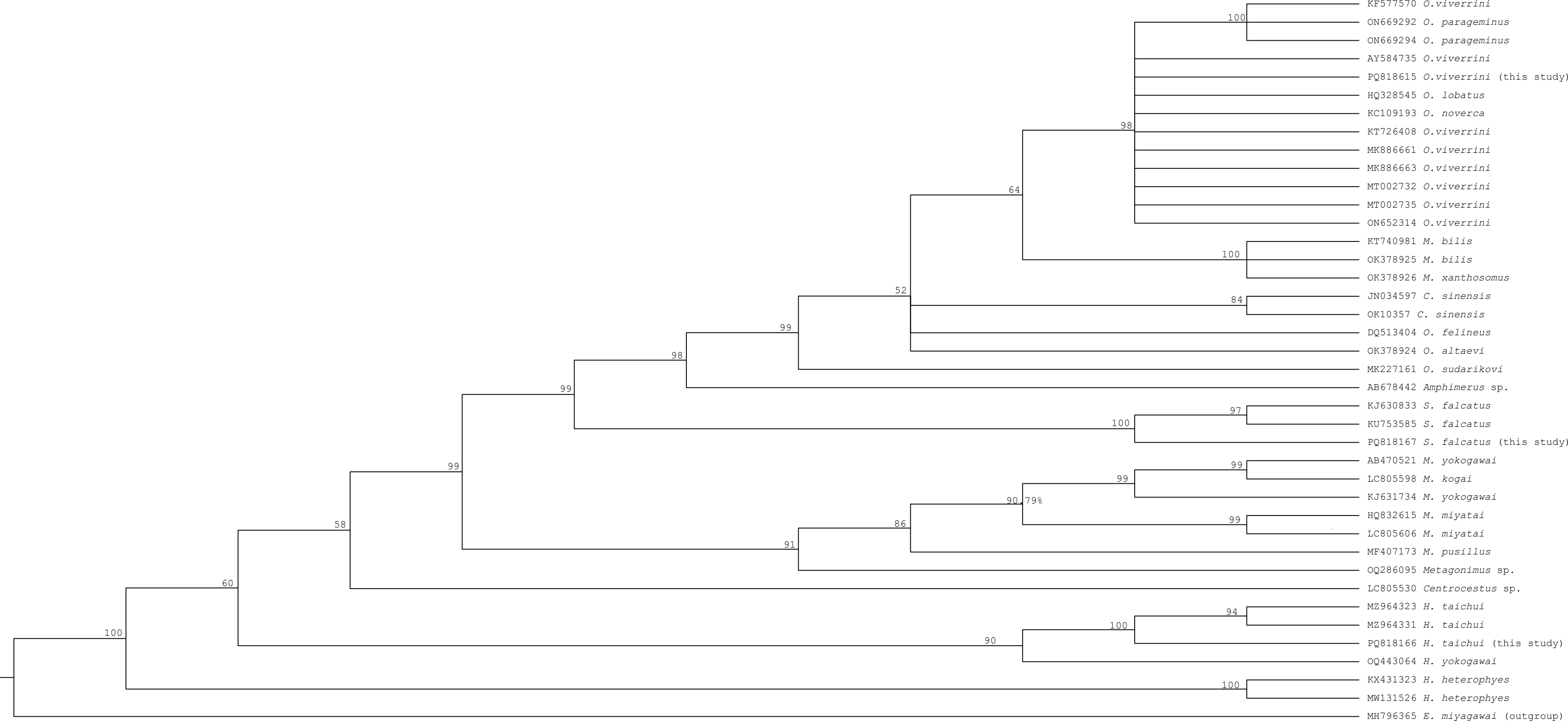

Supplement: Supplementary file 1 — Supplementary material [file mmc1.pdf]
